# Preliminary Study on Electrochemical Deposition of Graphene on Steel Substrate via In Situ Oxidation Using Cyclic Voltammetry

**DOI:** 10.3390/ma18112440

**Published:** 2025-05-23

**Authors:** Mattia Pelucchi, Brigida Alfano, Giuseppe Cesare Lama, Raphael Palucci Rosa, Marina Cabrini

**Affiliations:** 1 Department of Engineering and Applied Sciences, University of Bergamo, 24044 Dalmine, Italy; raphael.rosa@unibg.it; 2 TERIN-FSD-DIN, ENEA—Italian National Agency for New Technologies, Energy and Sustainable Economic Development, R.C. ENEA, 80055 Portici, Italy; 3 Institute of Polymers, Composites and Biomaterials, National Research Council (IPCB-CNR), 80055 Portici, Italy; giuseppecesare.lama@cnr.it; 4 National Interuniversity Consortium of Materials Science and Technology (INSTM), 50121 Firenze, Italy; 5 Research Unit of Bergamo, CSGI—Centre of Colloid and Surface Science, 22044 Dalmine, Italy

**Keywords:** graphene coating, cyclic voltammetry deposition, in situ oxidation of graphene

## Abstract

This study explores an innovative method for depositing graphene directly onto metal surfaces, using cyclic voltammetry with a suspension of graphene in water. Most electrochemical deposition techniques up to now have concentrated on graphene oxide (GO) rather than pure graphene, largely because GO disperses more readily in water. This characteristic makes GO simpler to manipulate and apply in deposition processes, giving it an advantage in terms of usability and practicality. We demonstrated that graphene can indeed be deposited onto metal surfaces using this innovative electrochemical approach. We conducted a thorough characterization of the resulting graphene deposits, employing advanced techniques, including interferometric microscopy, scanning electron microscopy (SEM), energy-dispersive X-ray spectroscopy (EDS), and Raman spectroscopy. These analyses provided us with insights into the structural, chemical, and morphological characteristics of the graphene deposits. This comparison allowed us to assess the strengths and potential improvements needed for this direct deposition method, as it offers a more sustainable and streamlined alternative to conventional GO-based processes. One important finding is that, while the quality of these direct graphene deposits has not yet reached the level of GO-based coatings, this new approach has some compelling advantages. Specifically, it is a simpler, more environmentally friendly process that could streamline production and reduce the environmental impact compared to traditional methods using GO.

## 1. Introduction

The use of hydrogen as an energy carrier with a low environmental impact is considered a cornerstone of the energy transition, so much so that the European Union has included hydrogen production among the necessary steps for the decarbonization of the continent by 2050 [1]. At present, electrolytic hydrogen production can be convenient for exploiting peaks of overproduction of electricity from renewable sources such as wind and solar power as an alternative to storage in chemical batteries [2,3]. The hydrogen thus produced is blended with natural gas and fed into the gas distribution network.

In the dossier ‘Blending hydrogen from electrolysis into the European gas grid’ published by the European Commission’s Joint Research Centre (JRC), aimed at analyzing the impact of a future hydrogen injection into the European gas grid, it is reported that the current natural gas distribution grids can immediately accommodate a 5% share of H_2_, while by 2030 this share could be increased to 20% [4]. The problems associated with the transport of hydrogen in natural gas distribution networks currently in use are due to the possibility of dissociation of the hydrogen molecule on pipe walls that are made of steel, followed by diffusion into the metallic material with the possibility of embrittlement occurring [5]. Various methods proposed to prevent hydrogen from entering metallic materials include the application of polymeric [6], metallic [7], and ceramic coatings [8,9,10].

The application of multilayer polymeric coatings, such as fusion-bonded epoxy (FBE), polyethylene (PE), and multilayer polypropylene (PP) systems, is a key part of state-of-the-art industrial practice for mitigating hydrogen-induced degradation in natural gas pipelines [11]. These coatings act as primary barriers, limiting contact between moisture and aggressive species (including molecular hydrogen and hydrogen sulfide) and the wall pipe [11,12]. Moreover, the use of metallic underlayers (e.g., zinc-rich primers) is sometimes a feature of improved adhesion and enhanced cathodic protection. However, traditional coatings often have limitations regarding long-term hydrogen impermeability, particularly under the elevated pressure and temperature conditions that may be present in future hydrogen-enriched gas networks. Interest in alternative barrier technologies, including nanostructured coatings and two-dimensional materials like graphene, has therefore increased, as these are aimed at providing enhanced protection against hydrogen permeation and associated embrittlement phenomena [13]. The use of graphene as a coating potentially capable of reducing hydrogen ingress into metals is of interest both for pipelines and also for small components like regulation valves, sensors, and measurement dispositive.

Graphene, a two-dimensional sheet of sp^2^-hybridized carbon atoms, has recently garnered significant attention due to its unique combination of electrical, mechanical, and optical properties. These exceptional characteristics make graphene a promising material for a broad range of applications, including electronics, sensors, composites, and corrosion protection [14,15]. In addition, graphene is also impermeable; its dense lattice structure forms an effective barrier that blocks even helium atoms. This remarkable property makes graphene an ideal candidate for applications requiring high levels of impermeability [16,17,18]. Nam et al. studied the applicability of graphene deposited by chemical vapor deposition (CVD) on copper to prevent hydrogen embrittlement, finding a marked improvement up to 25% deformation of the copper [19]. The protective effect was attributed to the formation of a C-H bond that blocks hydrogen from entering the metal [19]. Shi et al. realized multilayer graphene deposits using ionic implantation on a nickel deposit, followed by high-temperature solubilization, demonstrating that the coating is able to decrease the corrosion rate of an X70 steel and slow down the diffusion of hydrogen [20].

However, these techniques require specific machinery capable of vacuuming the chamber where the process takes place and controlling the rate of evaporation and deposition of carbon on the substrate to give graphene. These techniques appear too slow and too expensive to be applied on a large and industrial scale for the protection of carbon steel in contact with hydrogen gas. Although the application of a graphene coating on pipes is not currently feasible, it could be performed on small components of complex shapes that would be difficult to coat with high vacuum techniques [21]. Currently, high-coverage deposits are primarily limited to small surface areas and typically require expensive chemical vapor deposition (CVD) processes [22,23]. Despite the recent attainment of CVD coatings measuring tens of millimeters squared [24], for larger surfaces the most reliable method involves the electroplating of chemically oxidized graphene (GO) because it is simpler, requires less equipment, and is more cost-effective [25,26,27]. Kim et al. [28] successfully deposited graphene on stainless steel using a GO solution, applying a constant potential of 10 V for 8 min. Their research aimed to evaluate the barrier effect of these deposits on hydrogen embrittlement, an important consideration in materials science. Similarly, Marcin Behunová et al. [29] achieved consistent and homogeneous graphene deposits on stainless steel specimens using the same voltage and a minimum duration of 8 min. Currently, graphene oxide (GO) is produced through the chemical oxidation of graphite using the Hummers method [24], which employs aggressive reagents that have a considerable environmental impact.

The aim of this work is to bypass the conventional step of converting graphite into graphene oxide (GO) through chemical oxidation. Instead, this method uses an electrochemical technique that enables in situ oxidation and subsequent rapid reduction directly on the metal surface. This approach simplifies the deposition process, making graphene coatings more accessible, environmentally sustainable, and well-suited for practical applications. Eliminating the need for chemical oxidation reduces both the complexity and environmental impact of graphene production, paving the way for broader use in industrial and anticorrosive coatings.

The relevance of this work lies in the fact that, in the literature, numerous articles address graphene deposition starting from chemically obtained graphene oxide and applying a constant potential [25,26,27,28,29], whereas only a few studies focus on direct electrochemical oxidation [30] and deposition of graphene.

## 2. Materials and Methods

### 2.1. Solutions

Two types of graphene-based solutions were utilized: the first was a solution of graphene oxide (GO) at a concentration of 1 mg/mL in distilled water, synthesized via the traditional Hummers method [31]. This solution will later be referred to as the “GO solution”. The second solution consisted of graphene flakes dispersed in a 50% mixture of distilled water and ethanol, at a concentration of 1 mg/mL, which will later be referred to as “GE solution”. This suspension required sonication and the addition of 2 g/L of sodium lauryl sulfate to reduce surface tension, thereby enhancing the stability and homogeneity of the suspension. To ensure dispersion and prevent the aggregation of graphene flakes, both solutions were subjected to a two-hour sonication period prior to every test.

Graphene flakes that were used for the GE solution were synthesized using the liquid phase exfoliation (LPE) technique. This technique involves dispersing graphite flakes in a hydro-alcoholic solution and then sonicating the solution, which induces mechanical shear forces that separate the graphene layers from the graphite structure, resulting in high-quality, few-layer graphene flakes [32]. Afterwards, graphene flakes were separated from the solvents using freeze-drying. Graphene flakes thus obtained will be later referred to as “GE powder” (graphene-exfoliated powder).

### 2.2. Specimens

Wire-cut specimens of API 5L X65 pipeline steel were obtained from a gas pipeline. This steel is commonly used in the piping industry, and its chemical composition is shown in Table 1. Steels with similar compositions should produce the same results in terms of graphene deposits as API 5L X65 because the latter is typically composed of low-alloy carbon steel. The specimens were cylindrical with a diameter of 15 mm and a thickness of 5 mm, which was required to enable the use of a specimen holder compatible with the standard ASTM G5 cell [33]. All specimens underwent a surface preparation protocol involving progressive polishing with abrasive papers up to 400 grit to achieve a uniform finish. To ensure the removal of organic contaminants, each specimen was subjected to triple sequential washing with soap, followed by immersion in acetone within an ultrasonic bath. Prior to deposition, steel surfaces were activated via brief pickling in inhibited hydrochloric acid to activate surface reactivity and eliminate any oxide layers.

### 2.3. Potentiostatic Deposition

**Figure 1:**
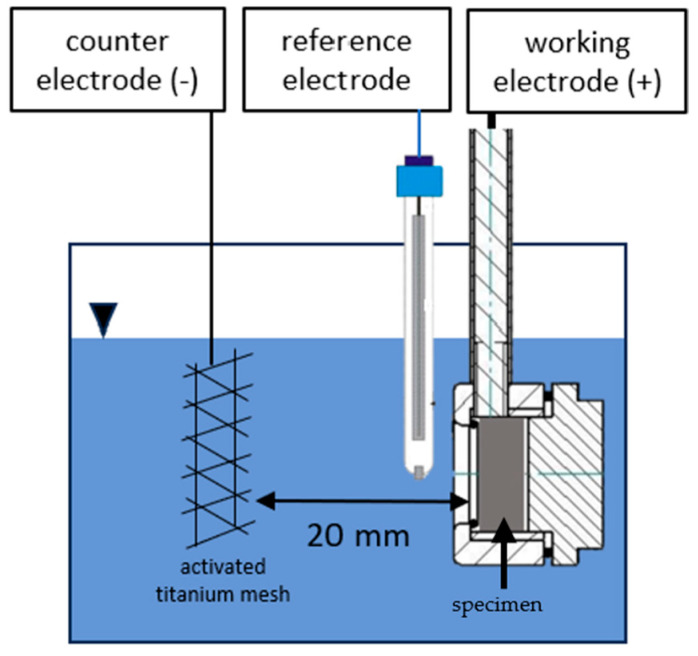
Potentiostatic cell setup.

This method involves the deposition of graphene and graphene oxide onto metal substrates through a controlled electrochemical process, applying a constant potential over a set period as described in previous studies [25,26,27,28,29]. The deposition process was carried out at room temperature within a glass electrochemical cell containing the specimen as the working electrode, an activated titanium counter electrode, and a AMEL 390/TCG saturated calomel reference electrode with a potential of +0.240 V vs. NHE at 25 °C (Figure 1). The reference electrode was positioned as close to the working electrode as possible to minimize ohmic drops in the solution. Throughout the experiments, the separation between the steel specimen and the counter electrode was maintained at 20 mm. A potential of 10 V was applied for a deposition duration of 8 min, with the specimen subjected to anodic polarization. The potentiostat employed for the experiments was an AMEL 7050 (AMEL s.r.l, Milan, Italy). These parameters align with those frequently cited in literature [28,29].

### 2.4. Cyclic Voltammetry Deposition

**Figure 2:**
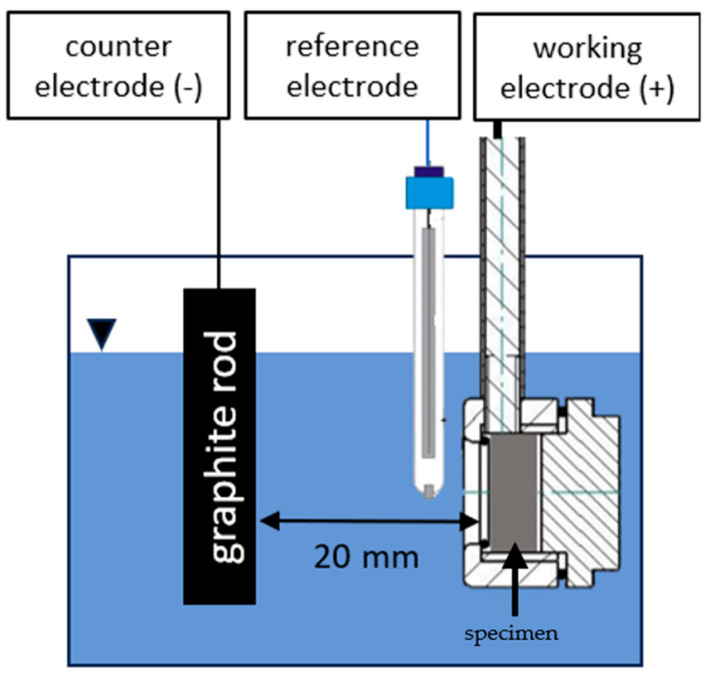
Cyclic voltammetry cell setup.

The graphene solution used in these tests was identical to that used in potentiostatic experiments (GE solution), following the same preparation methodology. To minimize corrosion of the metallic substrate, the solution pH, initially at approximately 5.5, was adjusted to pH 13 by adding sodium hydroxide, as further detailed in the discussion section. The specimens utilized for the cyclic voltammetry tests were identical to those prepared for the potentiostatic deposition, having undergone identical surface preparation procedures. This ensured comparability of the results across both experimental setups. The deposition was conducted at room temperature using a classic three-electrode voltammetry setup [34,35]. In this configuration, the working electrode was the specimen, the counter electrode was a graphite rod, and the reference electrode was an AMEL 390/TCG saturated calomel electrode with a potential of +0.240 V vs. NHE at 25 °C. The reference electrode was positioned as close to the working electrode as possible to minimize ohmic drops in the solution. Throughout the experiments, the separation between the steel specimen and the counter electrode was maintained at 20 mm (Figure 2). During these tests, the parameters investigated included the applied potential range and the number of cycles. The potentiostat employed for the experiments was an AMEL 7050 (AMEL s.r.l, Italy), programmed to perform cyclic voltammetry at a scanning rate of 50 mV/s, varying the potential according to a triangular waveform across a specified range depending on the test conditions. Following the deposition process, the specimen was rinsed with distilled water and subsequently dried in a laboratory dryer before undergoing analysis.

### 2.5. Coating Characterization Methods

The deposits were initially characterized by being observed under an optical microscope. The surface of deposits, which appeared to be best in terms of uniformity and degree of coating, was reconstructed using an interferometric microscope to assess the surface roughness and to evaluate the shape and depth of any craters. These deposits were also observed using a SEM equipped with an EDX probe. Samples were also analyzed using a Raman spectroscopy system.

### 2.6. Instruments and Software Used

The digital microscope utilized in this study was a KEYHENCE VHX-7000 (KEYHENCE ITALIA spa, Milan, Italy). The interferometric microscope utilized in this study was a SENSOFAR SNEOX (Sensofar, Terrassa, Spain) interferometric microscope. SEM analyses were done using a ZEISS SIGMA 300 (Carl Zeiss S.p.A., Milan, Italy) field emission scanning electron microscope (FESEM) equipped with an OXFORD XACT energy-dispersive x-ray spectroscopy (EDX) probe. The parameters utilized for the acquisition of SEM photographs are specified in the caption of each image. The EDX spectra were produced using a beam acceleration voltage of 8 keV, as the heaviest element in the specimens analyzed was iron.

Samples were also analyzed using a HORIBA XploRA PLUS Raman spectroscopy system (HORIBA ITALIA SRL, Rome, Italy) with a 532 nm laser; the acquired spectra are the result of 10 accumulations, performed in backscattering configuration. In order to avoid overheating the deposit, the laser was used at 1% of its maximum power (*P*_max_  =  20 mW).

The data were processed and subsequently represented in the graphs of this article using an electronic spreadsheet, i.e., Excel.

## 3. Results and Discussion

### 3.1. GE Powder

**Figure 3:**
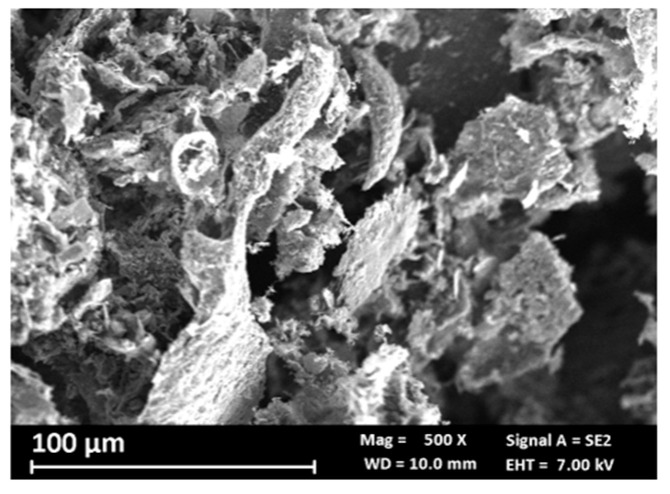
SEM images at 500× magnification: GE powder.

Before the dispersion, graphene flakes (GE powder) were analyzed using scanning electron microscopy (SEM) and energy-dispersive X-ray (EDX). GE powder is made of particles of different shapes and sizes and is not homogeneous (Figure 3); EDX analysis shows that it is composed essentially of carbon (95% atomic) and some oxygen (3% atomic); traces of aluminum and silicon are also present (Table 2). The carbon to oxygen ratio is very high (~31.7), and the presence of impurities such as Al and Si is probably due to the production process.


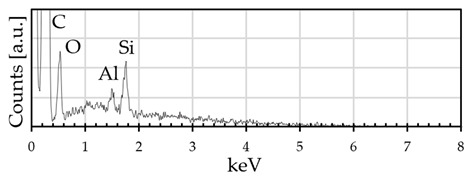


### 3.2. Potentiostatic

The morphology of the resulting graphene coatings was examined and characterized using optical and scanning electron microscopy (SEM), while energy-dispersive X-ray (EDX) analysis was employed to assess the elemental composition of the coatings (Table 3).

**Figure 4:**
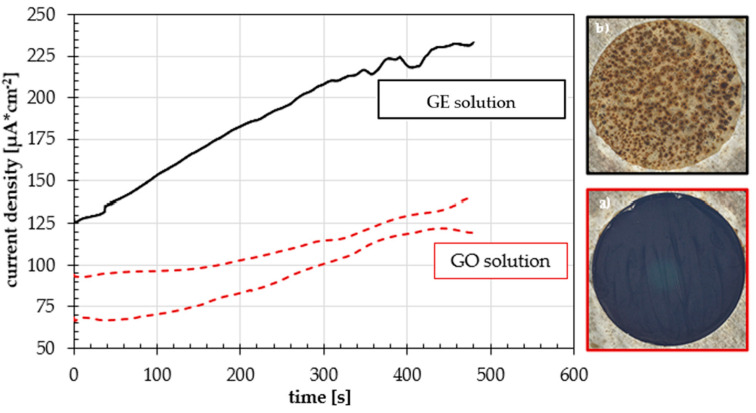
Current flowing between the test piece and the counter electrode in the GO solution and in the GE solution, and the surface of the specimens after a potentiostatic polarization of 10 V for 8 min in different solutions: (**a**) GO solution, and (**b**) GE solution.

The deposits obtained at the end of polarization and the graph that shows the trend of the current during deposition are shown in Figure 4. The GO solution consistently results in the formation of good deposits, whereas the GE solution, conversely, rarely deposits, and when it does, the deposit is typically insignificant and dissolves rapidly.

Anodic currents increase as time passes. The conductivity of the two solutions is different and is about 66 kΩ/cm for the GO solution and about 80 kΩ/cm for the GE solution. The deposition obtained with chemically oxidized GO is much more homogeneous and covers the metallic substrate better than that obtained from the solution of unreduced graphene. According to the theory [25,26,27,36], negatively charged GO was deposited on the anode, while deposition was not observed on cathodes. In the absence of voltage, no visible film was formed on all electrodes.

From the digital microscope observation also shown in the figure, it can be observed that the deposit obtained with the GO solution has a high degree of coating and appears homogeneous, while the deposit obtained from the GE solution is inhomogeneous and has a very low degree of coating. Since the deposit obtained from the GE solution is very poor while that obtained from the GO solution is much better, a method of oxidizing the graphene contained in the GE solution by electrochemical means in order to create a solution of oxidized graphene more like the GO solution was sought.

Pristine graphene does not deposit through a constant-potential electrochemical process due to its unique structure and lack of reactive functional groups. Graphene oxide, however, due to its oxygen-containing functional groups, is more responsive to electrochemical methods.

### 3.3. Cyclic Voltammetry

Accordingly to the previous statement, graphene was deposited under variable potential tests, employing cyclic voltammetry. This approach involves cycling the applied potential over a defined range, promoting periodic adsorption and reduction processes at the electrode interface. This method thus provides a more suitable pathway for graphene deposition, overcoming the limitations associated with constant potential methods.

Cyclic voltammetry between 0 and +6 V was made in the GE solution (non-oxidized graphene solution) to study the evolution of the voltammetric curve. In order to be able to highlight the peaks related to graphene reactions, any other electrochemical contributions of the system must be excluded, i.e., the oxygen reaction evolution (OER) and the peaks of oxidation and growth of iron oxide in alkaline solution. For this reason, voltammetric curves obtained on the same material in the presence and absence of graphene in solution at the same pH were compared.

**Figure 5:**
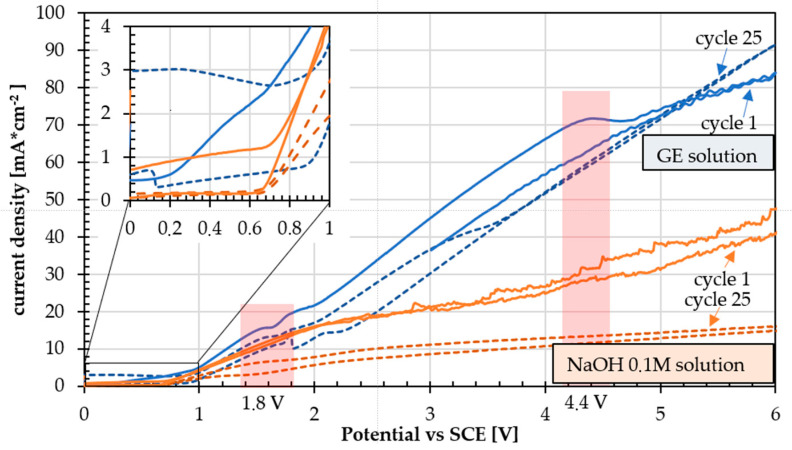
Comparison of series of voltammetry in graphene and NaOH 0.1 M solution from 0 to 6 V on API 5P X65 specimens.

In Figure 5, the comparison between the first and last cycle of the series of voltammetries for the GE solution (blue) and for NaOH solution 0.1 M (orange) in the potential range between 0 and +6 V is shown.

**Figure 6:**
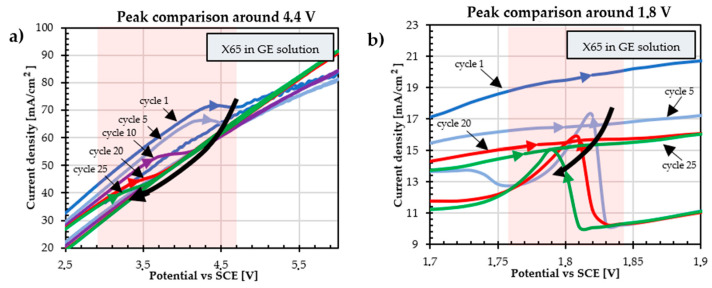
Comparison of GE solution peaks on API 5L X65 specimens: (**a**) around 4.4 V, and (**b**) around 1.8 V.

In the range of water stability (up to about 0.6 V vs. SCE), the steel surface of the specimens is in a passive state [37], so the anodic current decreases as the number of voltammetric cycles increases. At high potentials, the curves become very disturbed, probably due to the oxygen bubbles effect. In graphene solution (GE solution), the anodic current is higher than that recorded in the NaOH solution alone and continues to increase after the onset of oxygen evolution, suggesting the involvement of a superimposed anodic process. During the anodic scan, there is a not well-defined broad peak around 4.4 V (Figure 6a): the peak current decreases with the increase in the number of voltammetry cycles, and the peak potential shifts to less anodic values. The areas corresponding to these peaks are highlighted with red stripes on the graph. The solution of NaOH 0.1 M does not present these peaks, confirming that it cannot be considered to represent electrochemical reactions involving iron ions only, since the same X65 specimens were used in both solutions and the oxidation of graphene.

Given that graphene oxidation is nonstoichiometric, it is not feasible to ascertain an oxidation potential, but the presence of a black deposit on the metal surface suggests the growth of a carbon-rich coating on the substrate.

The presence of the small peaks in the return scan in the potential range around 1.8 V vs. SCE (Figure 6b) seems to indicate a partial reduction of the coatings, which could finally be formed by a mix of graphene and graphene oxide.

The possibility of having coatings on steel using cyclic voltammetry was further investigated by applying different voltammetry cycles to the specimens, varying the number of cycles and the potential range.

### 3.4. Effect of the Number of Cycles on the Coating

**Figure 7:**
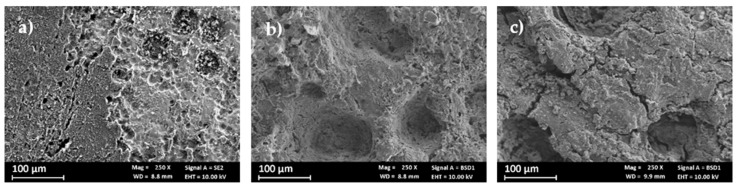
Surface of the specimen coated with CV at different numbers of cycles: (**a**) 10 cycles, (**b**) 25 cycles, and (**c**) 100 cycles.

Depositions were made using 10, 25, and 100 cycles of voltammetry at a pH of 13. The 10-cycle tests were conducted to ensure a sufficient number of specimens were available for analysis within a reasonable timeframe (approximately 30 min per deposit). The 25-cycle and 100-cycle tests were conducted to evaluate the trend in deposit quality with respect to the number of voltammetric cycles. The observation of these deposits at SEM (Figure 7) shows the poor quality and low degree of coating of the 10-cycle deposits, whereas the 100-cycle deposits are thick and cover the whole surface but tend to fracture, a characteristic which is detrimental to a coating. The coatings obtained with 25 cycles are much more homogeneous than those obtained with 10 cycles, and there do not appear to be large cracks on the surface, as in the case of 100 cycles. Therefore, it was decided to continue the deposition by keeping the number of voltammetry cycles at 25.

**Figure 8:**
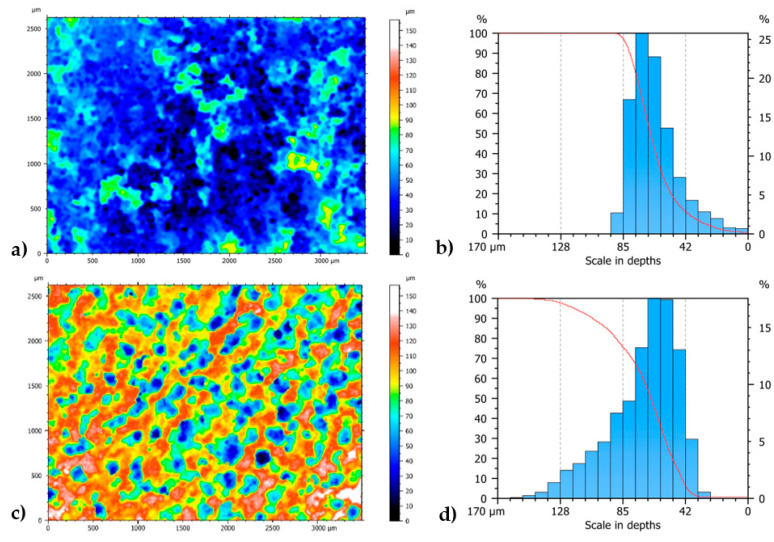
Surface reconstruction of the specimen coated with CV at different numbers of cycles: (**a**) 25 cycles, (**b**) 25 cycles’ Abbott diagram, (**c**) 100 cycles, and (**d**) 100 cycles’ Abbott diagram.

Further confirmation of the better quality of the deposits obtained with 25 cycles compared to those obtained at 100 is provided by the analysis of their surface roughness using an interferometric microscope (Figure 8). The absorption coefficient of the coating is high because the graphene absorbs a lot of visible light, and this makes the process of optimizing the survey parameters more complex. The analyses were carried out on both the 100-cycle and 25-cycle specimens at different magnifications. From the analyses, Abbot diagrams were obtained, which show the distribution of the height of the reconstructed surface. The 25-cycle specimen histogram has a narrower base than the 100-cycle specimen, indicating that the average surface of the 25-cycle specimen is less uneven. Profiles from the surface analyzed reveal craters in both deposits. 

### 3.5. Effect of Potential Ranges

**Figure 9:**
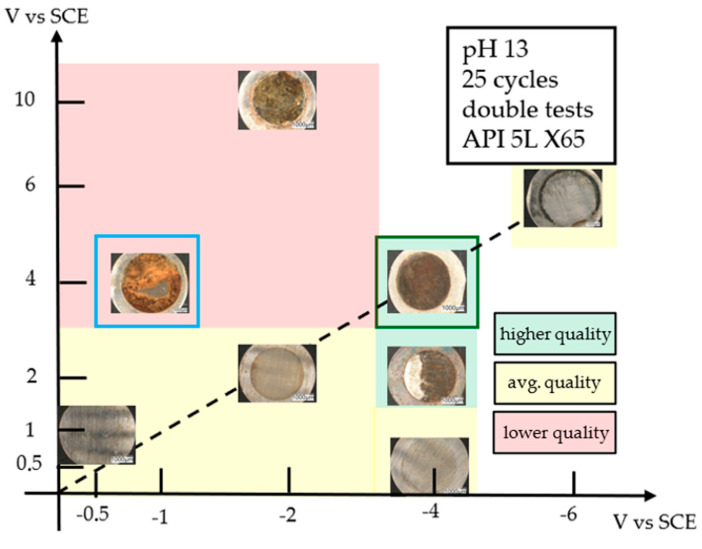
Surface areas of the deposits obtained as a function of the potential range scanned using CV with coating evaluation.

The graph in Figure 9 shows the potential ranges of the cyclic voltammetry carried out on the X65 specimens.

**Figure 10:**
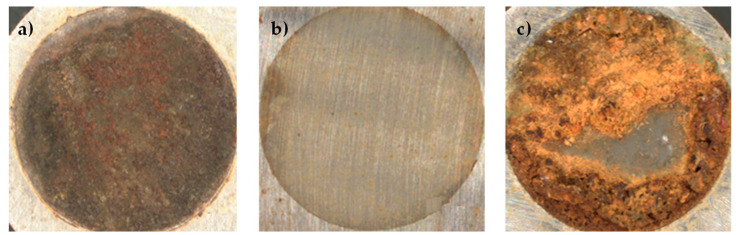
Surface of the deposits obtained after 25 voltammetry cycles at different potentials: (**a**) −4/+4 V, (**b**) −1/+4 V, and (**c**) −2/+2 V.

**Figure 11:**
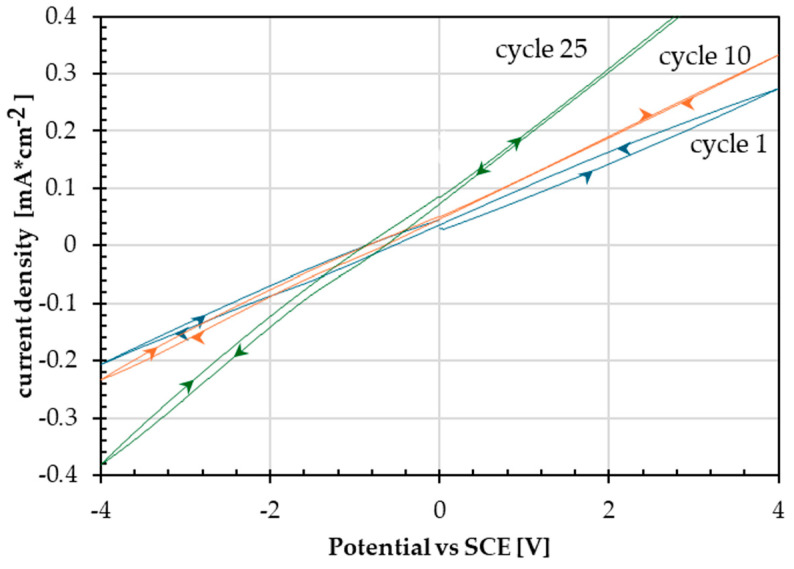
Comparison of first and last cyclic voltammetry cycles in GE solution from −4 to +4 V on API 5P X65.

The deposit that appears to be most uniform and with the highest degree of coating is the one highlighted in the dark green box, obtained by scanning the potential range between −4 and +4 V (Figure 10a). Figure 11 shows the first, the tenth, and the last cyclic voltammetry cycles that produced this deposit.

Deposits in the red area have an excessive amount of iron oxide that prevents the formation of a homogeneous graphene deposit. The deposits in the yellow area show the presence of a layer of graphene on the surface that appears homogeneously distributed over the whole surface, but its thickness is very thin and EDX surveys reveal the presence of large quantities of iron oxide and therefore it is not clear how much of this deposit is composed of graphene (Figure 10b). Deposits in the green area are those with consistent deposit thickness and a good degree of coating. Special mention should be made of the deposition between −1 and +4 V (blue box) because it shows a uniform graphene deposit but only in a restricted area of the specimen, while the rest of the surface is covered with a thick layer of iron oxide (Figure 10c).

### 3.6. Coating Characterization

Using as a reference the deposit obtained with 25 cycles of voltammetry between −4 and +4 V, analyses were made to investigate its chemical composition. From now on, this deposit will be referred to as a CV deposit, and the deposit obtained from the potentiostatic applied to the GO solution will be reported as the GO deposit.

**Figure 12:**
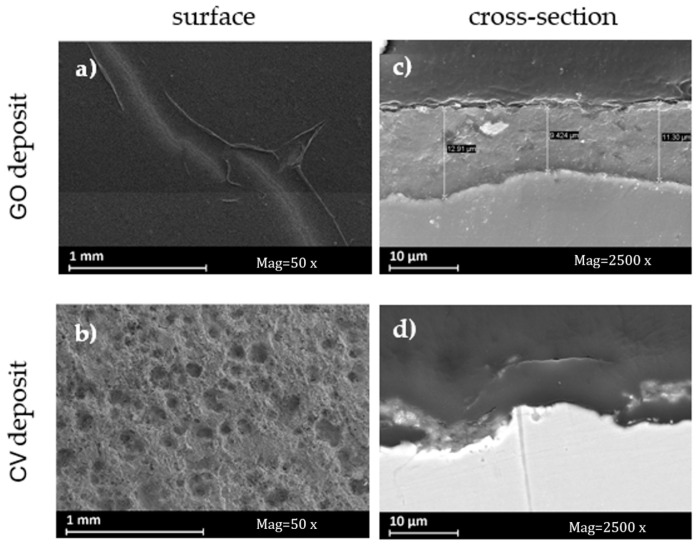
SEM images of the surface and cross section of (**a**,**c**) GO deposit and (**b**,**d**) CV deposit.

SEM observations of these deposits show a clear difference in coating quality. GO deposit appears visually more homogeneous and less wrinkled, while the surface of the CV deposit is strewn with craters (Figure 12). Their thickness was observed by sectioning coated specimens embedded in resin. The GO deposit has an average thickness of about 10 microns, and the coating seems uniform and completely covers the observed surface. The CV deposit, on the other hand, has some areas with a thickness of around 5 microns, and other areas have a thin coating or probably no coating at all. The deterioration of the steel specimen surface due to the anodic polarization of the specimen during cyclic voltammetry can be observed when the steel specimen is looked at in sections. Nevertheless, this polarization is imperative for the oxidation of the oxidized graphene. The deposits analyzed in this study were obtained through polarization in an aqueous solution. It is probable that polarization in other environments might reduce the corrosion rate of the metal substrate. Tests to this effect will be the subject of a future paper.

**Figure 13:**
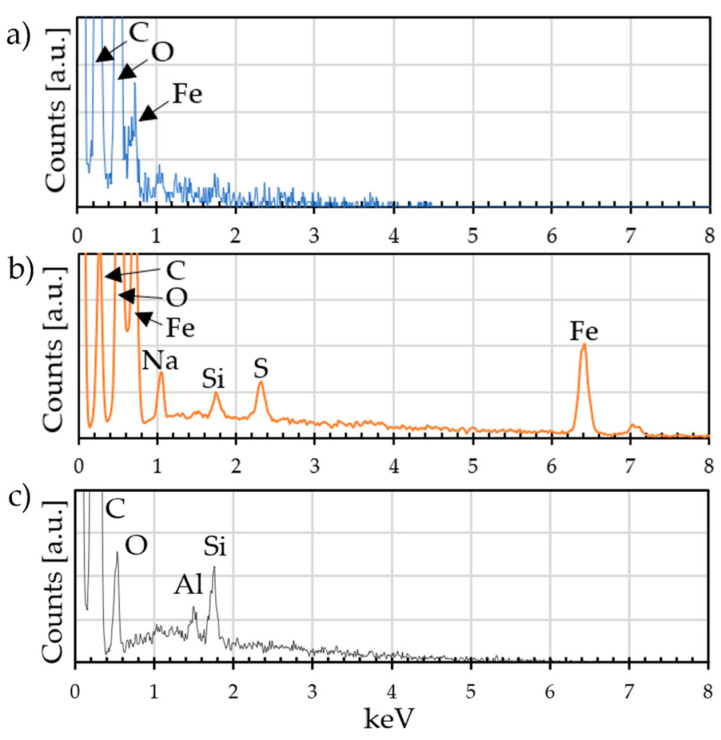
EDX spectra of surface composition of (**a**) GO deposit, (**b**) CV deposit, and (**c**) graphene powder.

The EDX analysis (Figure 13) shows a difference between the two deposits. The GO deposit is 50% carbon and 50% oxygen, while the CV deposit is a combination of carbon, iron, and oxygen. The presence of carbon indicates the formation of a graphene-based deposit on the metal substrate, the presence of iron and oxygen indicates the formation of iron oxide and oxidized graphene, while sodium and sulfur are detected due to the addition of sodium laurylsulphate in solution. EDX compositions are shown in Table 3.

**Figure 14:**
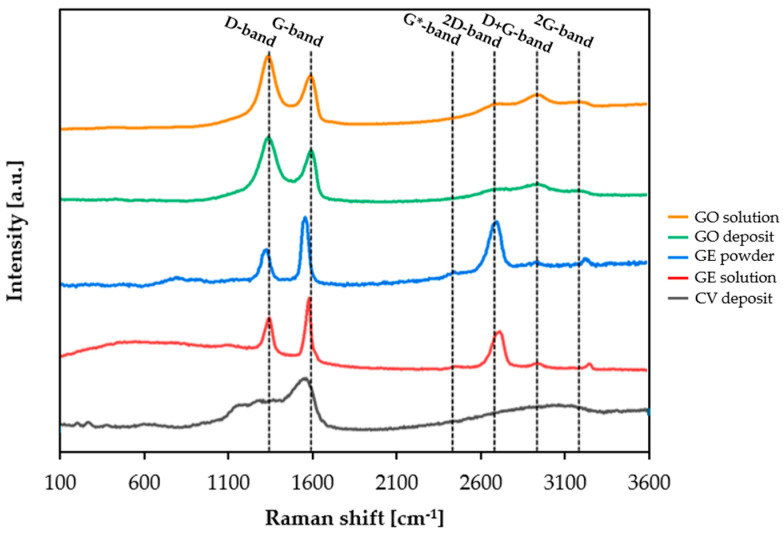
Raman spectra of GO solution, GO deposit, Ge powder, GE solution, and CV deposit (532 nm laser).

The deposits were also analyzed using Raman spectroscopy. This technique was used to establish whether the structures of both graphene and graphene oxide were somehow altered during the two respective selected electrochemical deposition processes. As a reference, both the GO and GE solutions were characterized by drop casting. Also, pristine GE powder was analyzed (Figure 14 and Table 4). As can be seen, the spectra of both the GO solution and GO deposit after potentiostatic polarization present practically the same peaks (D-, G-, 2D, D + G- and 2G- bands), with the same shape and position. Moreover, the ratio between the intensities of D-band respect to G-band (I_D_/I_G_), useful to evaluate the degree of order of the material (the lower it is, the closer is the sample to a graphene-like arrangement– sp^2^ carbon; the higher it is, the more is the amount of sp^3^ hybridized carbon), results only slightly reduced. This could be ascribed to a partial modification of the graphene oxide structure, upon potentiostatic deposition, becoming, most likely, moderately amorphous [38]. On the other hand, when GE powder is compared with GE solution, some evident differences arise. In particular, the position of D-band and G-band resulted in both shifting to higher values, meaning that the graphene layer reduced its thickness upon dispersion in water/ethanol solution [39], with an increased degree of disorder (in this case, I_D_/I_G_ has passed from 0.65 to 0.79). The most evident change the GE material had experienced was due to the cyclic voltammetry deposition. With this method, the Raman spectra (CV deposit) revealed an amorphous material with a certain amount of sp^2^ carbon kept upon deposition [40,41,42,43,44,45], which guarantees the electrical conductivity when graphitic material with mixed sp^2^–sp^3^ hybridization is used [42]. This analysis corroborates the hypothesis that during cyclic voltammetry, graphene is oxidized to GO, which is then deposited on the surface of the metal specimen. This deposit is different from that obtained by the potentiostatic deposition of pure GO, because during the cathodic scan of cyclic voltammetry, a partial reduction of GO can take place, forming the mix sp^2^-sp^3^ amorphous deposit, like the one obtained using the potentiostatic deposition of GO.

The influence of the chemical composition of CV deposits (in particular, the presence of electrochemically produced iron oxide) on their ability to decrease hydrogen permeation should have been evaluated using tests according to the Devanathan–Stachurski method but both coatings suffer from cathodic disbonding. For this reason, other investigation techniques are being evaluated.

## 4. Conclusions

The aim of this study was to bypass the conventional step of converting graphite into graphene oxide (GO) through chemical oxidation and to utilize an electrochemical technique that enables in situ oxidation and subsequent rapid reduction directly on the metal surface. The principal findings of this study are:

- The potentiostatic deposition technique allows for the formation of uniform, smooth graphene oxide (GO) coatings with poor adhesion. However, this method is not suitable for the deposition of non-oxidized graphene.
- The deposition of graphene on a metal surface by direct electrochemical in situ oxidation and subsequent reduction via cyclic voltammetry represents a novel technique that has yet to be documented in the existing literature.
- Raman analyses have confirmed the presence of graphene, which was oxidized prior to reduction by cyclic voltammetry and deposited at the surface.
- SEM, EDX, and interferometric observations of the deposits show discontinuities, both in terms of morphology and composition, that can be attributed to the deterioration of the substrate, which has resulted in a reduction in the quality of the coating.

Although the deposits obtained with graphene oxidized using the cyclic voltammetry method do not yet have the same quality as those obtained using chemically oxidized graphene, this technique seems to have great potential both from the point of view of resources and time required to oxidize graphene and from the point of view of environmental impact. In the future, the cyclic voltammetry parameters will be optimized to improve the quality of the deposits, and once a homogeneous and uniform deposit is obtained, permeation tests will be carried out using the Devanathan–Stachurski method to evaluate the barrier effect of these coatings. During these tests, the problem of cathodic disbonding will have to be considered. To complete this study, this method will also be used to compare and evaluate the effectiveness of the barrier effect produced by the three types of coatings mentioned in this work (graphene DLC, GO deposit, and CV deposit). This will allow the evaluation of which factors could play a role in the quality and imperviousness of the coatings (e.g., high presence of iron oxide) and which can be optimized to improve the final characteristics of the coatings.

A critical consideration in the implementation of graphene coatings for the protection of hydrogen-exposed components concerns the potential release of chemisorbed gas. As demonstrated in the existing literature, the hydrogenation of graphene—through the formation of covalent C-H bonds—is a reversible process that can be activated thermally or by other energetic stimuli. As demonstrated by Elias et al. [46], hydrogen can be desorbed from hydrogenated graphene (graphane) through thermal treatment, effectively restoring the original graphene structure. However, the energy required to break the covalent C–H bond in graphene is estimated to be in the range of 2.5–3.0 eV per hydrogen atom. According to the Arrhenius equation, this energy corresponds to desorption temperatures in the range of approximately 300–500 °C [47,48]. Simulations conducted by Zhou et al. [49] further support this interpretation, indicating that at 300 °C the C–H bond remains thermally stable under standard conditions. As a result of the above considerations, we might assume that the release of hydrogen accumulated in the graphene coating under ambient conditions is unlikely. On the other hand, in confined environments such as pipelines or reservoirs, such a release, although probably negligible, could lead to a danger of H_2_ accumulation in explosive concentrations during maintenance operations as a result of oxygen entering the pipeline and this possibility suggests the need to consider the dynamic behavior of adsorbed hydrogen on graphene coatings in future studies and in the engineering of graphene coatings for applications in hydrogen-containing environments.

## Figures and Tables

**Table 1:** Mass fraction composition of API 5L X65 steel.

Weight %	C max	Mn max	P max	S max
API 5L X65	1.45	0.03	0.03	0.03

**Table 2:** EDX surface composition and EDX spectra of GE powder.

Atomic %	C	O	Al	Si
Graphene powder	95	3	traces	traces

**Table 3:** EDX surface composition of GO deposit, CV deposit, and graphene powder.

Atomic %	C	O	Fe	S	Na	Al	Si
GO deposit	50	50	traces	/	/	/	/
CV deposit	30	41	26	1	2	/	/
Graphene powder	95	3	/	/	/	traces	traces

**Table 4:** Raman results of the analyzed samples.

Sample	D-Position	G-Position	I_D_/I_G_
GO solution	1349.61	1601.83	1.30
GO deposit	1349.14	1601.43	1.22
GE powder	1336.83	1565.71	0.65
GE solution	1355.71	1589.99	0.79
CV deposit	1319.75	1571.78	0.71

## Data Availability

The raw data supporting the conclusions of this article will be made available by the authors on request.
